# Does distance from a clinic and poverty impact visit adherence for noncommunicable diseases? A retrospective cohort study using electronic medical records in rural Haiti

**DOI:** 10.1186/s12889-020-09652-y

**Published:** 2020-10-14

**Authors:** Lily D. Yan, Dufens Pierre-Louis, Benito D. Isaac, Waking Jean-Baptiste, Serge Vertilus, Darius Fenelon, Lisa R. Hirschhorn, Patricia L. Hibberd, Emelia J. Benjamin, Gene Bukhman, Gene F. Kwan

**Affiliations:** 1 Boston Medical Center, 72 East Concord St., Boston, MA 02118 USA; 2 Zanmi Lasante, Cange, Haiti; 3 Hôpital Universitaire de Mirebalais, Mirebalais, Haiti; 4 Feinberg School of Medicine, Northwestern University, Chicago, IL USA; 5 Boston University School of Medicine, Boston, MA USA; 6 Boston University School of Public Health, Boston, MA USA; 7 Partners In Health, Boston, MA USA; 8 Harvard Medical School, Boston, MA USA; 9 Brigham and Women’s Hospital, Boston, MA USA

**Keywords:** Noncommunicable disease, Global health, Visit adherence, Retention in care, Haiti

## Abstract

**Background:**

Adherence to regular outpatient visits is vital to managing noncommunicable diseases (NCDs), a growing burden in low and middle-income countries. We characterized visit adherence among patients with NCDs in rural Haiti, hypothesizing higher poverty and distance from the clinic were associated with lower adherence.

**Methods:**

We analyzed electronic medical records from a cohort of adults in an NCD clinic in Mirebalais, Haiti (April 2013 to June 2016). Visit adherence was: 1) visit constancy (≥1 visit every 3 months), 2) no gaps in care (> 60 days between visits), 3) ≥1 visit in the last quarter, and 4) ≥6 visits per year. We incorporated an adapted measure of intensity of multidimensional poverty. We calculated distance from clinic as Euclidean distance or self-reported transit time. We used multivariable logistic regressions to assess the association between poverty, distance, and visit adherence.

**Results:**

We included 463 adult patients, mean age 57.8 years (SE 2.2), and 72.4% women. Over half of patients had at least one visit per quarter (58.1%), but a minority (19.6%) had no gaps between visits. Seventy percent of patients had a visit in the last quarter, and 73.9% made at least 6 visits per year. Only 9.9% of patients met all adherence criteria. In regression models, poverty was not associated with any adherence measures, and distance was only associated with visit in the last quarter (OR 0.87, 95% CI [0.78 to 0.98], *p* = 0.03) after adjusting for age, sex, and hardship financing.

**Conclusions:**

Visit adherence was low in this sample of adult patients presenting to a NCD Clinic in Haiti. Multidimensional poverty and distance from clinic were not associated with visit adherence measures among patients seen in the clinic, except for visit in the last quarter. Future research should focus on identifying and addressing barriers to visit adherence.

## Background

Noncommunicable diseases (NCDs) including hypertension, diabetes, and cardiovascular disease are rapidly rising in Haiti and other low and middle-income countries (LMICs) [1–5]. In 2018, NCDs accounted for an estimated 60.6% of all deaths in LMICs, and 48.3% of all disability adjusted life years [6]. The sustainable development goals call for both quality of care and equity to reduce premature mortality from NCDs, and financial risk protection to alleviate NCDs’ economic impact [7]. In Haiti, the prevalence of chronic health conditions is high. The nationally representative 2016 Demographic and Health Survey (DHS) found overweight prevalence among 35–64 year old adults at 44% in women, 18% in men, hypertension at 49% in women, 38% in men, and diabetes at 14% in women, 8% in men [8]. A community survey in select urban and rural communes found similar rates of chronic diseases, except for a lower rate of hypertension prevalence at 15.6% [9]. Patients with more severe NCD conditions tend to present late and have poorer outcomes. In the urban Hôpital de l’Université d’État, 98.8% of heart failure patients presented with high severity disease (New York Heart Association class III or IV), and had high in-patient mortality at 23.3% [10]. At the rural Hôpital Universitaire de Mirebalais (HUM), 37% of adult medical admissions are due to heart failure, with an in-hospital mortality of 12% [11].

Visit adherence, also known as retention in care, is an important precondition for successful chronic disease management in LMIC where most health systems require patients to come to the facility to obtain medications [12–14]. Low visit adherence thus implies low medication adherence. At HUM, only 36% of patients with heart failure who survived a primary hospitalization had linkage to outpatient care with a followup visit within 30 days of discharge, suggesting the vast majority did not continue to receive guideline-directed therapy [11]. Visit adherence has been variously defined as number of missed visits, proportion of kept visits, number of regular intervals (e.g. 3 months) with at least one visit, and gaps between visits [12]. Various patient and healthcare system factors have been associated with retention in care, including degree of symptoms, financial constraints, distance from the clinic, decreased wait times, and improved patient satisfaction [15–17]. While routinely measured in the HIV/AIDS care continuum literature and linked to viral loads [18, 19], medication adherence, and mortality [20, 21], visit adherence and its predictors have not been routinely examined for chronic disease care in LMIC.

To address the gap in understanding chronic disease care retention, we examined the patterns of visit adherence using several measures in a NCD clinic in rural Mirebalais, Haiti. We hypothesized that higher multidimensional poverty and higher distance from the clinic were associated with lower visit adherence.

## Methods

### Design, setting

We conducted a retrospective cohort study at the noncommunicable disease clinic (NCD Clinic) at the Hôpital Universitaire de Mirebalais (HUM) in the rural central plateau of Haiti, where there were only about eight doctors per 100,000 population during the study period [22]. HUM is a 300-bed public academic referral hospital, created in partnership between the Haitian Ministry of Public and Population Health, the non-governmental organizations Zanmi Lasante (Haiti based), and Partners In Health (Boston based). It provides over 123,000 outpatient clinic visits annually and is the primary healthcare center for the 165,000 people who live closest to the hospital. At the time of the study, patients paid only a one-time registration fee of about 50 Haitian Gourdes (about US $1), and all subsequent clinic visits, hospitalizations, diagnostic tests, and medications were provided free. Demographic data and clinic visits are recorded in an electronic medical record (EMR) (OpenMRS, Grandville, Michigan, USA). Socioeconomic data was collected and transcribed to REDCap, a secure, web-based software platform [23].

### Participants

In our retrospective cohort study we included all adults aged 18 years and older presenting to the NCD Clinic with socioeconomic and clinical data in the EMR. The NCD Clinic cares for patients with chronic conditions including hypertension, diabetes, heart failure, or stroke. This analysis is limited to the chronic NCDs that are within the scope of the clinic. Our study included visits between April 2, 2013 to June 30, 2016. We restricted our analysis to adults whose first visit occurred between April 2, 2013 and June 30, 2015 to allow for at least 1 year of follow-up (SFigure 1).

### Data sources and management

Study authors did not have direct access to the EMR and contacted hospital information technology staff to extract a de-identified database of patients meeting inclusion criteria. We exported demographic data, multidimensional poverty indicators, patient addresses at the 3rd subnational level (communes) from registration data, and exported all visit dates from the NCD clinic data. These two databases were merged at the person-level (SFigure 1).

Duplicate medical record numbers were manually examined, and records with more complete clinical data were kept. Multiple patient visits with the same date were manually examined to determine if they resulted from accidental system duplication (same time stamps and clinical data) or from multiple visits in the same day (different time stamps, different clinical data). Accidental duplicates were removed.

### Outcomes

The primary outcome of interest was visit adherence using four definitions adapted from Poles et al. adapted to the HUM clinical context: 1) visit constancy, 2) no gaps in care, 3) visit in the last quarter, 4) at least six visits a year, and a summary measure of yes on all four metrics [24]. 1) Visit constancy was defined as at least one visit every 3 months (calendar quarters) in the first year after initial visit. We adapted the definition of 2) no gaps in care as no more than 60 days between visits, based on previous research and the fact that prescriptions in the NCD Clinic are usually given for a maximum of 60-day periods and outpatient visits are meant to be at least once every 2 months. 3) Visit in the last quarter was at least one visit between April 1, 2016 and June 30, 2016. 4) The metric of at least six visits per year was adapted and defined as having at least six visits per year, calculated by taking the total number of visits divided by length of time between first visit and June 30, 2016. Lastly, a summary measure of “yes” on all four metrics was calculated. All variables were dichotomous. The length of potential follow-up time after the first visit was at least 1 year, but varied based on when the patient first presented.

### Variables

The independent variables of interest were distance from the clinic and poverty. A patient’s distance from the clinic was calculated as the Euclidean distance from their commune’s centroid, extracted from the EMR, to the clinic location in kilometers using the Near Distance Spatial Analyst tool in ArcMap 10.6.1 (Esri, Redlands, CA). Euclidean distance was represented as a continuous variable in 10-km increments. To define poverty, we used a two-step process. First, we calculated the intensity of individual multidimensional poverty [25], and second we categorized people as poor if their intensity of multidimensional poverty was greater than the median value for the entire NCD Clinic (poor compared to their peers).

We incorporated multidimensional poverty adapted from standardized measures developed by the Oxford Poverty and Human Development Initiative and the United Nations Development Programme [26, 27]. It traditionally uses ten indicators among three dimensions: health (child mortality, nutrition), education (years of schooling, attendance), and living standards (cooking fuel, sanitation, water, electricity, floor material, and assets). We examined the nine indicators that were collected as part of the NCD Clinic registration process in a subset of patients – excluding electricity which was not collected (STable 1). Each indicator has defined thresholds to be considered deprived (yes/no). While multidimensional poverty can be calculated as a continuous index at a national level using Demographic and Health Survey data to account for both prevalence and intensity of poverty across the poor in a geographic area, for our individual level data we calculated poverty as the percent of collected variables that were deprived for each person—or, the intensity of multidimensional poverty. A more detailed description of our process and poverty measures are described elsewhere [25].

We also measured poverty through hardship financing: having sold possessions or borrowed money to pay for medical expenses [28]. Other covariates included sex (woman vs. man), age in 10-year increments as a continuous variable, and year of first visit.

### Statistical analysis

For visit adherence measures, we analyzed with counts and frequencies. To test the association between independent variables with visit adherence measures, we first conducted univariate analysis with single variable logistic regression for multidimensional poverty index, hardship financing, and distance from clinic. Then, we preformed age and sex adjusted logistic regressions for each of these three variables. Lastly, we included year of first visit into the multivariable logistic regressions. Robust standard errors were used throughout. Patients with missing data for independent or dependent variables were excluded from all analyses.

For sensitivity analyses, we used patient reported transit time instead of Euclidean distance to facility (< 1 h, ≥1 h), used only poverty or only hardship financing instead of both to account for collinearity, and included disease severity into the regressions. We created three disease categories in descending order of severity: congestive heart failure, patients with either type 1 diabetes or type 2 diabetes on insulin, and other. Patients were categorized based on their most severe condition if they had multiple (e.g. patient categorized as heart failure if they had both heart failure and diabetes).

Maps were created using ArcMap 10.6.1, using shapefiles of Haiti downloaded from Humanitarian Data Exchange. Clinic patients were plotted in their communal sections.

### Ethical approval

The ethical review boards of Zanmi Lasante (ZLRC ID #3) and Boston University Medical Center (H-34326) approved the study. As only de-identified routinely collected clinic data were used, the need for informed consent was waived by both review boards. The RECORD statement checklist is available in the supplement.

### Patient and public involvement

The research question was informed by anecdotal reports by patients around their challenges in attending regular visits at the NCD Clinic. Patients and the public were not otherwise involved in the design, recruitment, conduct, or analysis of this study. As there was no intervention and the study only used routinely collected medical data, we did not assess the additional burden of the study on patients.

## Results

### Study population

We included 463 patients who had their first visit between April 2, 2013 and June 30, 2015, with any follow-up visit through June 30, 2016, for a total of 8841 outpatient visits (SFigure 1). Seventy-two percent of patients were women, and the sex-pooled mean age was 57.8 years, with a mean age of 56.9 years for women and 61.2 years for men (Table 1). Using the median number of multidimensional poverty indicators deprived (deprived in 2 out of 9 indicators) as the threshold, 62% of patients were considered poor, with a higher proportion of women (67.7%) than men (48.1%). There was a similar, though smaller, sex difference in hardship financing (68.1% women vs 59.8% men).

**Table 1:** Characteristics of adult patients presenting to NCD Clinic in rural Haiti, 2013–2016

	Men (*N* = 128)		Women (*N* = 335)		Total (*N* = 463)	
	Percent	SE	Percent	SE	Percent	SE
**Sex**	27.6	2.1	72.4	2.1	–	–
**Age**, yrs. (mean, SE)	61.2	4.4	56.9	2.6	57.8	2.2
**Poor** (deprived > 2/9 indicators)	48.1	4.9	67.7	2.9	62.0	2.5
**Hardship financing**	59.8	5.4	68.1	3.2	65.7	2.8
**Euclidean distance**, km (median, IQR)	11.4	(9.3–36.3)	9.9	(9.1–35.5)	11.0	(9.1–36.3)
**Travel Time**	–	–	–	–	–	–
< 30 min	36.4	5.1	43.8	3.4	41.6	2.8
30 min-1 h	25.0	4.6	18.4	2.6	20.3	2.3
1-2 h	22.7	4.5	25.3	3.0	24.6	2.5
2-3 h	12.5	3.5	9.2	2.0	10.2	1.7
3-6 h	2.3	1.6	1.8	0.9	2.0	0.8
Unknown	1.1	1.1	1.4	0.8	1.3	0.7
**Disease Category**	–	–	–	–	–	–
Hypertension	12.5	2.9	15.2	2.0	14.5	1.6
Diabetes	22.7	3.7	14.3	1.9	16.6	1.7
Heart failure	4.7	1.9	3.0	0.9	3.5	0.8
Multiple	25.0	3.8	28.4	2.5	27.4	2.1
Other	3.1	1.5	4.5	1.1	4.1	0.9

Legend: *SE* standard error

The median Euclidean distance was 11.0 km overall (IQR 9.1–36.3 km). In self-reported travel time, most patients traveled less than 30 min (43.8% women, 36.4% men) and almost all traveled less than 3 h. About a quarter of people had multiple chronic health conditions (28.4% women, 25.0% men). Hypertension diagnoses were similar across sexes (15.2% women, 12.5% men), however diabetes was lower in women (14.3%) then men (22.7%).

### Visit adherence measures

**Fig. 1:**
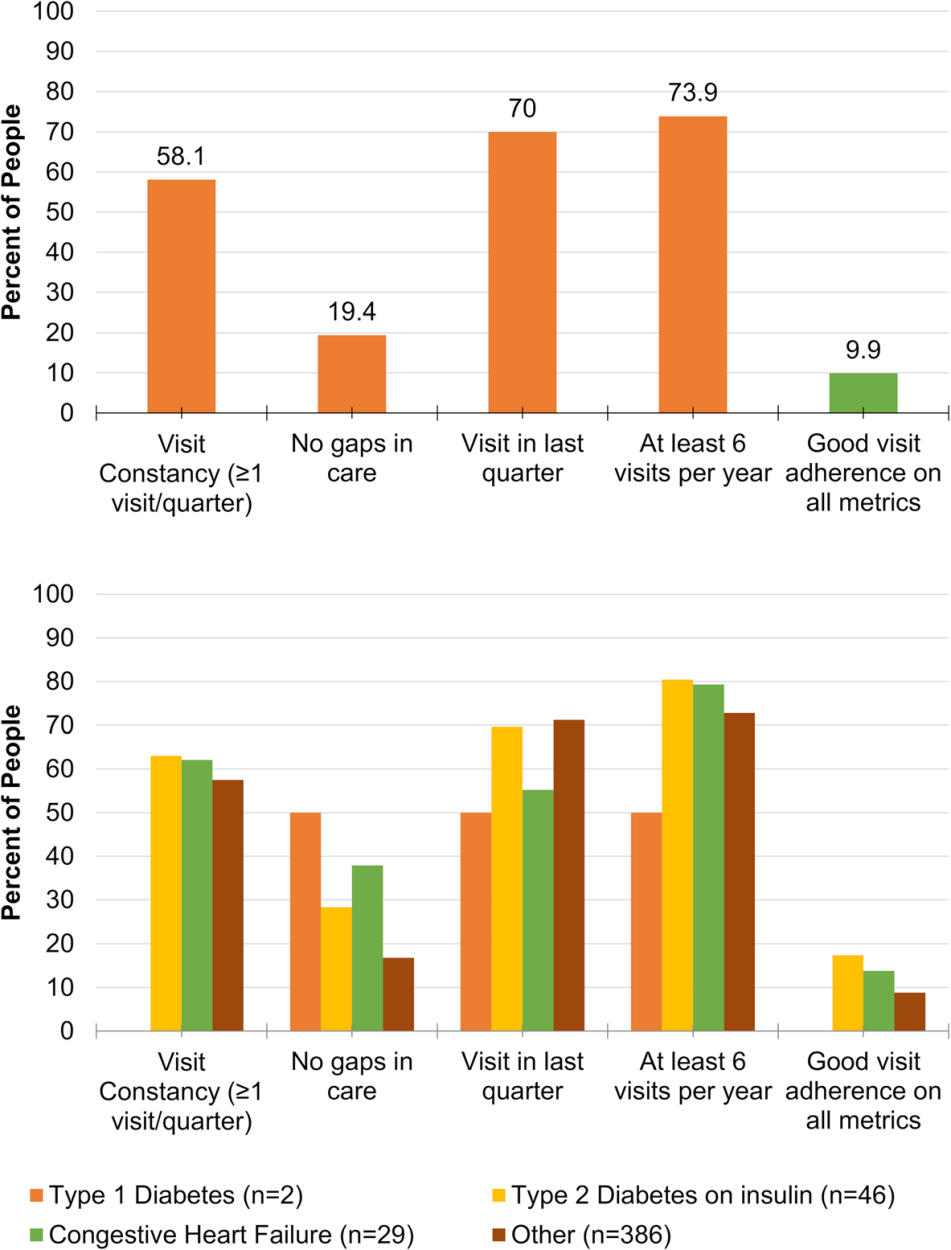
Visit adherence measures among patients in an NCD Clinic in rural Haiti. Proportion of patients who met criteria for each visit adherence measure. Top panel shows visit adherence for total NCD clinic patients. Bottom panel shows visit adherence by disease category

Visit adherence was varied across measures. About 58% of people had visit constancy (at least one visit every 3 months) (Fig. 1a). High proportions had a visit in the last quarter (70%) or at least six visits per year (73.9%). Adjusting for the length of time patients were in the analytic period (time from first clinic visit), the visits per year ranged from 0.42 to 19.9, with an interquartile range of 8 to 11.7.

The visit adherence measure with the lowest achievement among patients was having no gaps in care of more than 60 days, with only 19.4% of patients meeting this measure. In sensitivity analyses using more lenient criteria, 44.7% of patients had no gaps in care of more than 90 days, and 58.8% of patients had no gaps more than 120 days (STable 2). Taken together, only 9.9% of patients achieved all measures of visit adherence.

**Fig. 2:**
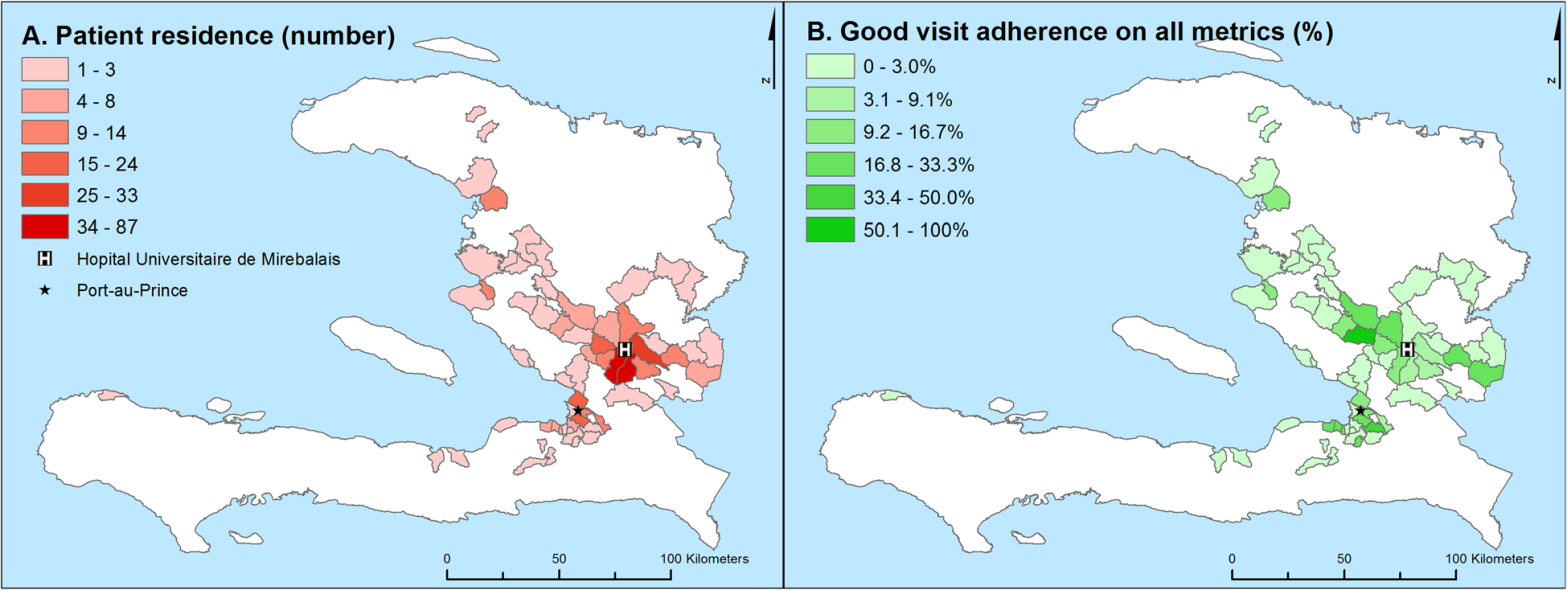
Density map of patient residence (A) and good visit adherence on all metrics (B). Darker shades indicate higher density of either patient residence (absolute numbers) or good visit adherence on all four metrics (percentage). The location of the capital Port-au-Prince, and the Hôpital Universitaire de Mirebalais are labeled

As patients with more severe diseases like insulin dependent diabetes or heart failure may have different patterns of retention than asymptomatic conditions like early diabetes or hypertension, we also examined visit adherence measures among the three most severe conditions: type 1 diabetes, type 2 diabetes on insulin, and heart failure (Fig. 2b). By disease severity, visit adherence measures were highest for people with type 2 diabetes on insulin with 17.4% achieving all measures (Fig. 1b).

### Associations between poverty, distance, disease, and visit adherence

Figure 2a illustrates where patients lived and Fig. 2b shows the proportion of patients in a given geographic zone that had good visit adherence on all metrics. While there is substantial heterogeneity, visit adherence measures seem to be slightly higher along a major road to the northwest of the hospital (Fig. 2b and SFigures 2A-D).

Univariate analysis revealed neither being multidimensionally poor nor hardship financing were associated with any of the visit adherence measures or the summary measure. A 10 km increase in Euclidean distance was associated with increased likelihood of no gaps in care (OR 1.13, 95% CI 1.03 to 1.23) and decreased likelihood of visits in last quarter (OR 0.91, 95% CI 0.83 to 0.99) (STable 3). In the age- and sex-adjusted multivariable logistic regressions, only Euclidean distance was associated with decreased likelihood of visits in the last quarter (OR 0.90, 95% CI 0.82 to 0.99) (STable 4).

**Fig. 3:**
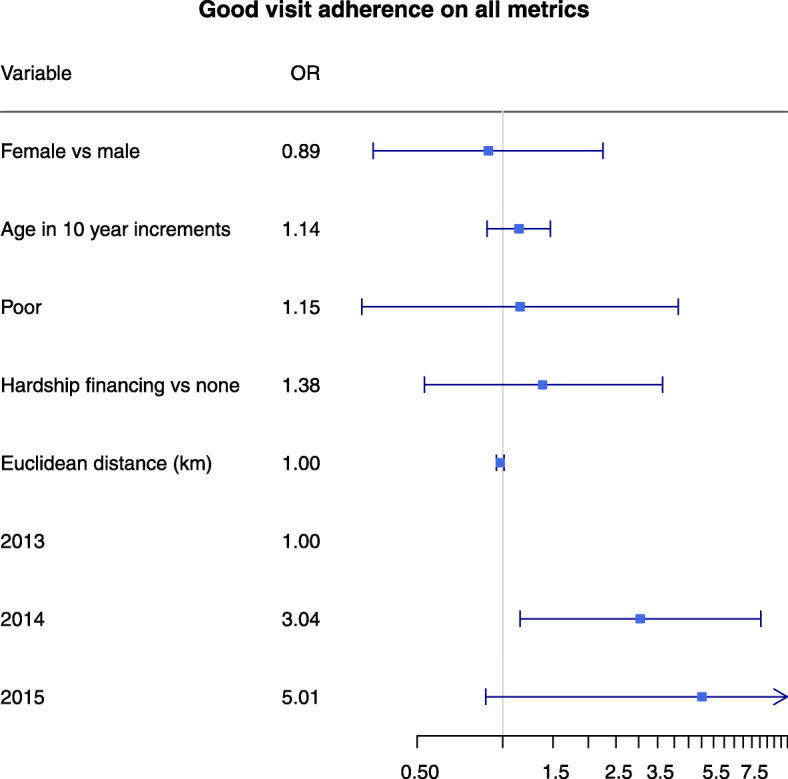
Odds ratios for factors related to the outcome “Good visit adherence on all metrics”. Odds ratios and 95% confidence intervals predicted from logistic regression are shown. Intervals that do not cross the vertical gray line at 1 are considered statistically significant with an alpha level of 0.05

In multivariable logistic regression, in general poverty and distance from the clinic were not associated with visit adherence measures (Table 2, Fig. 3, SFigure 3). Neither being multidimensionally poor nor hardship financing was significantly associated with the outcomes of visit constancy, no gaps in care, visit in the last quarter, or at least 6 visits per year. A 10 km higher mean Euclidean distance had an inverse association only with having a visit in the last quarter (OR 0.87, 95% CI 0.78 to 0.98).

**Table 2:** Multivariable logistic regression on poverty and distance factors associated with visit adherence measures in an NCD Clinic in rural Haiti

	Visit Constancy (*n* = 287)				No Gaps in Care (*n* = 285)				Visit in Last Quarter (*n* = 287)				At least 6 visits per year (n = 287)				Yes on all metrics (n = 287)			
Characteristic	OR	95% CI		*p* value	OR	95% CI		p value	OR	95% CI		p value	OR	95% CI		p value	OR	95% CI		p value
Female vs male	0.85	0.49	1.48	0.56	0.78	0.38	1.60	0.49	**2.29**	**1.29**	**4.05**	0.005*	**2.00**	**1.12**	**3.57**	0.02*	0.89	0.35	2.25	0.81
Age (10 yr increments)	1.10	0.93	1.31	0.27	**1.31**	**1.05**	**1.64**	0.02*	**0.71**	**0.59**	**0.86**	< 0.001*	**0.80**	**0.65**	**0.99**	0.04*	1.14	0.88	1.47	0.31
Poor	1.15	0.64	2.07	0.64	1.42	0.60	3.38	0.43	1.04	0.56	1.91	0.90	1.09	0.56	2.11	0.80	1.15	0.32	4.13	0.83
Hardship financing vs none	1.11	0.65	1.87	0.70	1.04	0.51	2.11	0.91	1.28	0.73	2.25	0.39	1.34	0.76	2.38	0.32	1.38	0.53	3.64	0.51
Euclidean distance (km)	1.00	0.99	1.01	0.72	1.01	1.00	1.02	0.17	**0.99**	**0.98**	**1.00**	0.03*	0.99	0.98	1.00	0.17	0.98	0.95	1.01	0.18
2013	1.00				1.00				1.00				1.00				1.00			
2014	0.77	0.46	1.29	0.31	**7.09**	**3.43**	**14.68**	< 0.001*	**0.34**	**0.19**	**0.59**	< 0.001*	**0.38**	**0.22**	**0.68**	0.004*	**3.04**	**1.15**	**8.06**	0.03*
2015	**5.04**	**1.12**	**22.73**	0.04*	3.43	0.77	15.31	0.11	1.07	0.31	3.68	0.91	4.02	0.51	31.83	0.19	5.01	0.87	28.91	0.07

Legend: Bold and asterisks indicate statistically significant *p* values < 0.05

Women, compared with men, had a higher likelihood of a visit in the last quarter (OR 2.29, 95% CI 1.29 to 4.05), and at least 6 visits per year (OR 2.00, 95% CI 1.12 to 3.57) (Table 2). Increasing age had different associations based on the visit adherence measure. A ten-year higher mean age was associated with increased likelihood of no gaps in care (OR 1.31; 95% CI 1.05 to 1.64), but reduced likelihood for visit in the last quarter (OR 0.71; 95% CI 0.59 to 0.86) and for having at least 6 visits a year (OR 0.80; 95% CI 0.65 to 0.99) (Table 2).

In sensitivity analyses, using patient reported transit time instead of Euclidean distance, longer transit time was associated with reduced likelihood of visit in the last quarter (OR 0.43, 95% CI 0.24 to 0.76) and at least 6 visits a year (OR 0.52, 95% CI 0.29 to 0.94) (Supplemental Table 5). In regression models only using intensity of multidimensional poverty (Supplemental Table 6), or hardship financing (Supplemental Table 7), neither poverty measure was associated with any of the visit adherence measures, and other associations remained consistent. Lastly, adjusting for disease severity did not change the associations between poverty, Euclidean distance, sex, or age on visit adherence measures, however patients with diabetes on insulin had a higher OR of good visit adherence (STable 8, STable 9).

## Discussion

In this study of patients presenting to an NCD clinic in rural Haiti, we found low visit adherence measured using several metrics: visit constancy, no gaps in care, visit in the last quarter, and at least 6 visits per year. Patients appeared to come in and out of care. Further distance to clinic was associated with lower likelihood of visit in the last quarter; the association was stronger for patient-reported travel time than Euclidean distance. Surprisingly, poverty and visit adherence measures were not associated. In prioritizing visit adherence measures, no gaps in care were likely the most important as they relate directly to medication adherence, but patients performed the worse on this measure.

Many factors have been associated with retention in care, including individual level factors (feeling sick and having symptoms, moral support from relatives, fear of stigma, misunderstanding about importance of care, financial constraints), provider level (negative healthcare provider attitudes, abusive language), health system level (overcrowding, inadequate resources), and contextual factors (proximity to clinic) [15–17]. Prior non-retention and tracing studies for HIV care to find patients who have been lost to follow-up have shown an inverse association between poverty and attendance at clinic [17, 29, 30]. In one study in South Africa, 34% of patients who had been lost to follow-up reported finances as a limiting factor [16]. Beyond inability to pay for transportation, poverty may influence nonadherence in other ways, such as competing priorities of work and childcare responsibilities [31], and lack of food that interferes with taking medications [32, 33].

The lack of association between poverty and visit adherence in our NCD sample could be explained by a number of factors. We have reported that patients presenting to the NCD clinic were not as poor as the surrounding community, perhaps leading to a narrower distribution of poverty among patients in the NCD clinic that limits statistical power [25]. Patients who presented to the NCD Clinic were also self-selected and engaged in care. We may have missed the poorest patients who did not present at all. Community-based data from the Haiti 2016–17 DHS relating socioeconomic status and distance to a health facility supports this presentation bias [8]. Considering wealth, more people in the poorest quintile lived more than 15 km from a health facility than in the richest quintile (41% vs 24%), suggesting that healthcare was still fairly centralized in poorer, rural areas. Efforts to improve visit adherence among people with chronic disease will likely require significant community outreach to bring the poorest people into clinic in the first place. Our NCD Clinic may also be inadvertently missing patients who choose to work and give less priority to their own chronic disease care and are unable to attend restrictive clinic hours. Beyond this, there may also be heterogeneity in the association with poverty by disease type that we could not detect due to small sample size, which deserves further study. Lastly, the user fee (although small) may still be a deterrent for the poorest to seek care at HUM.

Our finding that longer transport times or distance from clinic were associated with lower performance on visit adherence measures among patients with chronic conditions is consistent with current literature. In a qualitative survey in Tanzania among people living with HIV, both patients and healthcare providers reported long distance and high transport costs as major barriers to patient attendance at clinic [15]. In a meta-analysis of patient reported barriers to antiretroviral therapy adherence, distance to clinics was a significant factor [34]. Even in a high-income country HIV cohort, patients who traveled more than 5 miles vs. those who traveled less than 5 miles had 30% lower retention in care and lower viral suppression [35]. Given this established relationship between increased distance and decreased attendance, decentralizing care through community health workers or group medication pickups should be explored.

Based on prior literature, one might anticipate higher mean age to be associated with higher likelihood of visit adherence measures [24], but this was only true for no gaps in care. Prior research also suggests better health outcomes in women vs. men, which was true for having a visit in the last quarter and at least six visits a year. In the HIV/AIDS literature, increasing age was associated with greater engagement in care in the United Kingdom [36]. In a Tanzanian cohort, higher mean age was associated with a lower likelihood of gaps > 60 days between visits, and a higher likelihood of a visit in the last quarter [24]. In an urban adolescent HIV cohort in Port-au-Prince, Haiti, while age was not associated with lost to follow-up, being female was actually associated with a higher attrition either before antiretroviral initiation or after [37].

There are scarce data on the impact of poor visit adherence on intermediate and long-term clinical outcomes like mortality. Most studies on either HIV or NCDs collect data on patients within a healthcare facility. Patients who do not interact with the clinic, or present to another facility for follow-up care, rarely have data collected for long-term outcomes. A national registry including vital status is not assembled in Haiti. One proposed analytic solution is to assess survival outcomes among people who are lost to care using community sampling methods [38, 39]. Prospective, population representative cohorts are only just beginning to be formed for NCD care in LMIC to help address this deficit.

There are limitations to this study. In this tertiary facility-based analysis, we were unable to capture outcomes for patients seeking care at other institutions. For the no gaps in care measure, using 60 days to define a gap (usual follow-up in NCD clinic) would falsely categorize patients who might have been given a 90-day follow-up (usual in internal medicine clinic) which affects a minority of patients. We were unable to link visit adherence with long-term clinical outcomes. Fourth, we were not able to determine the ultimate outcome of patients lost to follow-up, and there was likely bias in this missing data (e.g. death vs. disengagement). Fifth, our distance measurements do not account for transitions between transportation modes (i.e., walking to bus station and waiting for public transit) and likely underestimated travel time. Sixth, patients sometimes arrived at the NCD clinic, but left without being registered or seen by a clinician due to long wait times. Thus, we may have underestimated visit adherence. Seventh, given our retrospective analysis of routinely collected data instead of separately collecting data specifically for research, our data may contain misclassification bias, missing data, and unmeasured confounding. Eighth, intensity of multidimensional poverty was originally developed for use with nationally representative data, while we have adapted it for use with hospital level data. Lastly, we only captured patient residence and multidimensional poverty once upon intake, and this may have changed during the study period.

## Conclusion

Low visit adherence for the measures of visit constancy, no gaps in care, visit in the last quarter, and at least 6 visits a year were seen for an NCD clinic in rural Haiti. There was no association between visit adherence and poverty, possibly due to the poorest patients never presenting to clinic. There was a small association with increased transport time and distance and worse visit adherence measures. These findings may be applicable to other low-income settings. Future work should focus on identifying barriers to care and understanding the intermittent patterns of care. Large, prospective longitudinal studies are needed to identify the relationship between poverty, distance, visit adherence, and clinical outcomes. It is important to improve visit adherence for patients with chronic conditions, to improve long-term clinical outcomes.

## Supplementary information

**Additional file 1: Supplemental Materials. Supplemental Tables 1–9 and Supplemental figures 1–3.** Supplemental Table 1 Adapted multidimensional poverty index. Supplemental Table 2: Alternative definitions of visit adherence measures. Supplemental Table 3: Univariate logistic regressions on factors associated with visit adherence measures in an NCD Clinic in rural Haiti. Supplemental Table 4: Sex and age adjusted multivariable logistic regressions for poverty and distance factors associated with visit adherence in an NCD Clinic in rural Haiti. Supplemental Table 5: Multivariable logistic regression on poverty and distance, using time travel instead of Euclidean distance, associated with visit adherence measures. Supplemental Table 6: Multivariable logistic regression on poverty and distance factors, with only MPI poverty, associated with visit adherence measures. Supplemental Table 7: Multivariable logistic regression on poverty and distance factors, with only hardship financing, associated with visit adherence measures. Supplemental Table 8: Multivariable logistic regression on disease severity, associated with visit adherence measures. Supplemental Table 9: Multivariable logistic regression on poverty and distance factors, adjusting for disease severity, associated with visit adherence measures. Supplemental Figure 1: Flow diagram of database linkage, and patient selection. Supplemental Figure 2: Density map of visit adherence measures. Supplemental Figure 3: Odds ratios for factors related to visit adherence measure outcomes.

**Additional file 2. **RECORD checklist. **RE**porting of studies **C**onducted using **O**bservational **R**outinely-collected **D**ata (**RECORD**) statement checklist.

## Data Availability

The data are not publicly available. The investigators requested and obtained the data from Zanmi Lasante and Hôpital Universitaire de Mirebalais. De-identified data are available upon reasonable request by contacting the Zanmi Lasante IRB at zlirb@pih.org.

## Abbreviations

DHS: Demographic and health survey
EMR: Electronic medical record
HUM: Hôpital Universitaire de Mirebalais
LMIC: Low- and middle-income countries
NCDs: Noncommunicable diseases
OR: Odds ratio
SE: Standard error
