# Supplementary material for: Does distance from a clinic and poverty impact visit adherence for noncommunicable diseases? A retrospective cohort study using electronic medical records in rural Haiti
Source: BMC Public Health. 2020 Oct 14;20:1545. doi: 10.1186/s12889-020-09652-y (PMC7556963; doi:10.1186/s12889-020-09652-y)
Supplement: Supplementary file 1 — Additional file 1: Supplemental Materials. Supplemental Tables 1–9 and Supplemental figures 1–3. Supplemental Table 1 Adapted multidimensional poverty index. Supplemental Table 2: Alternative definitions of visit adherence measures. Supplemental Table 3: Univariate logistic regressions on factors associated with visit adherence measures in an NCD Clinic in rural Haiti. Supplemental Table 4: Sex and age adjusted multivariable logistic regressions for poverty and distance factors associated with visit adherence in an NCD Clinic in rural Haiti. Supplemental Table 5: Multivariable logistic regression on poverty and distance, using time travel instead of Euclidean distance, associated with visit adherence measures. Supplemental Table 6: Multivariable logistic regression on poverty and distance factors, with only MPI poverty, associated with visit adherence measures. Supplemental Table 7: Multivariable logistic regression on poverty and distance factors, with only hardship financing, associated with visit adherence measures. Supplemental Table 8: Multivariable logistic regression on disease severity, associated with visit adherence measures. Supplemental Table 9: Multivariable logistic regression on poverty and distance factors, adjusting for disease severity, associated with visit adherence measures. Supplemental Figure 1: Flow diagram of database linkage, and patient selection. Supplemental Figure 2: Density map of visit adherence measures. Supplemental Figure 3: Odds ratios for factors related to visit adherence measure outcomes. [file 12889_2020_9652_MOESM1_ESM.docx]

**Does distance from a clinic and poverty impact visit adherence for noncommunicable diseases? A retrospective cohort study using electronic medical records in rural Haiti**

**SUPPLEMENTAL TABLES AND FIGURES**

Table of Contents

Supplemental Table 1: Adapted multidimensional poverty index 2

Supplemental Table 2: Alternative definitions of visit adherence measures 3

Supplemental Table 3: Univariate logistic regressions on factors associated with visit adherence measures in an NCD Clinic in rural Haiti. 4

Supplemental Table 4: Sex and age adjusted multivariable logistic regressions for poverty and distance factors associated with visit adherence in an NCD Clinic in rural Haiti. 5

Supplemental Table 5: Multivariable logistic regression on poverty and distance, using time travel instead of Euclidean distance, associated with visit adherence measures. 6

Supplemental Table 6: Multivariable logistic regression on poverty and distance factors, with only MPI poverty, associated with visit adherence measures. 7

Supplemental Table 7: Multivariable logistic regression on poverty and distance factors, with only hardship financing, associated with visit adherence measures. 8

Supplemental Table 8: Multivariable logistic regression on disease severity, associated with visit adherence measures. 9

Supplemental Table 9: Multivariable logistic regression on poverty and distance factors, adjusting for disease severity, associated with visit adherence measures. 10

Supplemental Figure 1: Flow diagram of database linkage, and patient selection 11

Supplemental Figure 2: Density map of visit adherence measures. 12

Supplemental Figure 3: Odds ratios for factors related to visit adherence measure outcomes. 13

# Supplemental Table 1: Adapted multidimensional poverty index

| **Dimension of poverty** | **Indicator** | **Deprived if…** | **Adaptation from OPHI definition** |
| --- | --- | --- | --- |
| Education | Years of schooling | No household member has completed 6 years of schooling | Patient or head of household has not completed 6 years of schooling |
| Education | Child school attendance | Any school aged child is not attending school up to class 8 | Any school aged child is not attending school |
| Health | Child mortality | Any child died in family in last 5 years | Any child died |
| Health | Nutrition | Any adult or child for whom there is nutritional information is stunted | Patient BMI < 18.5 kg/m2 |
| Standard of living | Sanitation | Household sanitation facility is not improved, or is shared with other households | Household toilet is not improved (open latrine, bush) |
| Standard of living | Drinking water | Household does not have access to safe drinking water | Household does not use improved water source (anything other than tap, wells, protected water source, rain, commercial water) |
| Standard of living | Floor | Household has dirt, sand, or dung floor | Household has dirt floor |
| Standard of living | Cooking fuel | Household cooks with dung, wood, or charcoal | Household cooks with wood or charcoal |
| Standard of living | Assess ownership | Household doesn’t have more than one of: radio, TV, telephone, bike, motorbike, refrigerator, and does not own car or truck | Household doesn’t have more than one of: radio, TV, telephone, refrigerator |

Legend: Electricity was excluded for the poverty assessment used in this study.

# Supplemental Table 2: Alternative definitions of visit adherence measures

| **Visit adherence measure** |  | **Frequency** | **Percent** |
| --- | --- | --- | --- |
| Visit constancy | Number of quarters with at least 1 visit within first year of initial visit |  |  |
|  | 0 | 12 | 2.6 |
|  | 1 | 42 | 9.1 |
|  | 2 | 58 | 12.5 |
|  | 3 | 82 | 17.7 |
|  | 4 | 269 | 58.1 |
| No gaps in care | no gap > 30 days | 11 | 2.4 |
|  | no gap > 60 days | 90 | 19.4 |
|  | no gap > 90 days | 207 | 44.7 |
|  | no gap > 120 days | 272 | 58.8 |
| Visit in last quarter | Yes | 324 | 70.0 |
| At least 6 visits per year | Yes | 342 | 73.9 |
| Yes to all | Yes | 46 | 9.9 |

# Supplemental Table 3: Univariate logistic regressions on factors associated with visit adherence measures in an NCD Clinic in rural Haiti.

|  | Visit Constancy | | | | No Gaps in Care | | | | Visit in Last Quarter | | | | At least 6 visits per year | | | | Yes on all metrics | | | |
| --- | --- | --- | --- | --- | --- | --- | --- | --- | --- | --- | --- | --- | --- | --- | --- | --- | --- | --- | --- | --- |
| Characteristic | OR | 95% CI | | p value | OR | 95% CI | | p value | OR | 95% CI | | p value | OR | 95% CI | | p value | OR | 95% CI | | p value |
| Female vs male | 0.85 | 0.56 | 1.29 | 0.44 | 0.81 | 0.49 | 1.34 | 0.42 | 1.69 | 1.10 | 2.60 | 0.02* | 1.85 | 1.18 | 2.88 | 0.01* | 0.77 | 0.40 | 1.48 | 0.43 |
| Age (10 yr increments) | 1.07 | 0.94 | 1.22 | 0.30 | 1.06 | 0.91 | 1.25 | 0.45 | 0.83 | 0.72 | 0.96 | 0.01* | 0.88 | 0.75 | 1.02 | 0.10 | 0.95 | 0.78 | 1.15 | 0.59 |
| Poor | 1.12 | 0.72 | 1.72 | 0.62 | 0.86 | 0.52 | 1.45 | 0.58 | 0.86 | 0.54 | 1.35 | 0.51 | 1.07 | 0.66 | 1.72 | 0.78 | 0.65 | 0.33 | 1.28 | 0.21 |
| Hardship financing vs none | 1.11 | 0.68 | 1.83 | 0.68 | 1.29 | 0.68 | 2.46 | 0.44 | 1.17 | 0.71 | 1.95 | 0.53 | 1.25 | 0.73 | 2.14 | 0.42 | 1.52 | 0.58 | 4.00 | 0.39 |
| Euclidean distance (10 km) | 1.05 | 0.94 | 1.17 | 0.41 | 1.13 | 1.03 | 1.23 | 0.01* | 0.91 | 0.83 | 0.99 | 0.03* | 0.96 | 0.88 | 1.06 | 0.43 | 0.98 | 0.87 | 1.11 | 0.80 |

Legend: statistically significant p values <0.05 are marked with an asterisk *

# Supplemental Table 4: Sex and age adjusted multivariable logistic regressions for poverty and distance factors associated with visit adherence in an NCD Clinic in rural Haiti.

|  | Visit Constancy | | | | No Gaps in Care | | | | Visit in Last Quarter | | | | At least 6 visits per year | | | | Yes on all metrics | | | |
| --- | --- | --- | --- | --- | --- | --- | --- | --- | --- | --- | --- | --- | --- | --- | --- | --- | --- | --- | --- | --- |
| Characteristic | OR | 95% CI | | p value | OR | 95% CI | | p value | OR | 95% CI | | p value | OR | 95% CI | | p value | OR | 95% CI | | p value |
| Female vs male | 0.78 | 0.48 | 1.28 | 0.33 | 0.81 | 0.45 | 1.47 | 0.49 | 1.64 | 1.00 | 2.71 | 0.050 | 1.67 | 1.00 | 2.78 | 0.05 | 0.63 | 0.31 | 1.26 | 0.19 |
| Age (10 yr increments) | 1.14 | 0.97 | 1.33 | 0.11 | 1.25 | 1.03 | 1.52 | 0.02 | 0.80 | 0.68 | 0.94 | 0.01 | 0.85 | 0.71 | 1.02 | 0.07 | 1.11 | 0.89 | 1.39 | 0.37 |
| Poor | 1.25 | 0.78 | 1.99 | 0.35 | 1.03 | 0.57 | 1.85 | 0.92 | 0.88 | 0.54 | 1.43 | 0.60 | 1.15 | 0.68 | 1.92 | 0.61 | 0.88 | 0.43 | 1.79 | 0.72 |
| 2013 | 1.00 |  |  |  | 1.00 |  |  |  | 1.00 |  |  |  | 1.00 |  |  |  | 1.00 |  |  |  |
| 2014 | 0.98 | 0.63 | 1.54 | 0.94 | 8.46 | 4.31 | 16.62 | 0.00 | 0.36 | 0.23 | 0.59 | 0.00 | 0.50 | 0.31 | 0.82 | 0.01 | 3.45 | 1.47 | 8.10 | 0.00 |
| 2015 | 6.32 | 2.22 | 18.03 | 0.00 | 8.98 | 3.40 | 23.71 | 0.00 | 1.14 | 0.43 | 3.04 | 0.79 | 2.65 | 0.76 | 9.17 | 0.12 | 8.47 | 2.82 | 25.50 | 0.00 |
| Female vs male | 0.88 | 0.51 | 1.51 | 0.63 | 0.84 | 0.41 | 1.71 | 0.64 | 2.21 | 1.26 | 3.87 | 0.01 | 2.00 | 1.13 | 3.54 | 0.020 | 0.89 | 0.34 | 2.30 | 0.81 |
| Age (10 yr increments) | 1.11 | 0.94 | 1.32 | 0.23 | 1.31 | 1.05 | 1.62 | 0.02 | 0.74 | 0.62 | 0.88 | 0.00 | 0.83 | 0.68 | 1.00 | 0.05 | 1.17 | 0.92 | 1.50 | 0.20 |
| Hardship financing vs none | 1.13 | 0.67 | 1.89 | 0.65 | 1.06 | 0.52 | 2.15 | 0.87 | 1.29 | 0.74 | 2.25 | 0.36 | 1.36 | 0.77 | 2.41 | 0.29 | 1.39 | 0.52 | 3.69 | 0.51 |
| 2013 | 1 |  |  |  | 1 |  |  |  | 1 |  |  |  | 1 |  |  |  | 1 |  |  |  |
| 2014 | 0.78 | 0.47 | 1.3 | 0.34 | 7.71 | 3.75 | 15.84 | 0 | 0.29 | 0.17 | 0.51 | 0 | 0.36 | 0.2 | 0.63 | 0 | 2.57 | 1 | 6.65 | 0.05 |
| 2015 | 4.98 | 1.12 | 22.18 | 0.03 | 3.56 | 0.86 | 14.84 | 0.08 | 0.77 | 0.24 | 2.55 | 0.67 | 3.31 | 0.43 | 25.56 | 0.25 | 2.94 | 0.56 | 15.38 | 0.2 |
| Female vs male | 0.82 | 0.51 | 1.32 | 0.41 | 0.8 | 0.45 | 1.44 | 0.46 | 1.64 | 1 | 2.71 | 0.05 | 1.74 | 1.04 | 2.88 | 0.03 | 0.64 | 0.31 | 1.31 | 0.22 |
| Age (10 yr increments) | 1.16 | 0.99 | 1.35 | 0.06 | 1.27 | 1.05 | 1.54 | 0.01 | 0.78 | 0.66 | 0.91 | 0 | 0.85 | 0.71 | 1.01 | 0.07 | 1.08 | 0.87 | 1.35 | 0.47 |
| Euclidean distance (10 km) | 1.03 | 0.93 | 1.14 | 0.58 | 1.05 | 0.95 | 1.17 | 0.33 | 0.90 | 0.82 | 0.99 | 0.04* | 0.95 | 0.86 | 1.05 | 0.31 | 0.86 | 0.71 | 1.03 | 0.10 |
| 2013 | 1 |  |  |  | 1 |  |  |  | 1 |  |  |  | 1 |  |  |  | 1 |  |  |  |
| 2014 | 0.94 | 0.59 | 1.49 | 0.79 | 8.02 | 4.06 | 15.87 | 0 | 0.41 | 0.25 | 0.66 | 0 | 0.53 | 0.32 | 0.88 | 0.01 | 3.99 | 1.69 | 9.45 | 0 |
| 2015 | 5.59 | 1.91 | 16.4 | 0 | 8.03 | 3.02 | 21.4 | 0 | 1.48 | 0.55 | 4.04 | 0.44 | 2.84 | 0.8 | 10.08 | 0.11 | 12.24 | 3.97 | 37.78 | 0 |

Legend: statistically significant p values <0.05 are marked with an asterisk *

# Supplemental Table 5: Multivariable logistic regression on poverty and distance, using time travel instead of Euclidean distance, associated with visit adherence measures.

|  | Visit Constancy | | | | No Gaps in Care | | | | Visit in Last Quarter | | | | At least 6 visits per year | | | | Yes on all metrics | | | |
| --- | --- | --- | --- | --- | --- | --- | --- | --- | --- | --- | --- | --- | --- | --- | --- | --- | --- | --- | --- | --- |
| Characteristic | OR | 95% CI | | p value | OR | 95% CI | | p value | OR | 95% CI | | p value | OR | 95% CI | | p value | OR | 95% CI | | p value |
| Female vs Male | 0.89 | 0.51 | 1.57 | 0.69 | 0.86 | 0.41 | 1.83 | 0.70 | 2.35 | 1.30 | 4.24 | 0.005* | 2.02 | 1.12 | 3.64 | 0.02* | 0.95 | 0.35 | 2.59 | 0.93 |
| Age (10 yr increments) | 1.12 | 0.94 | 1.34 | 0.21 | 1.30 | 1.02 | 1.65 | 0.03* | 0.71 | 0.58 | 0.86 | <0.001* | 0.80 | 0.65 | 1.00 | 0.05* | 1.17 | 0.89 | 1.54 | 0.27 |
| Poor | 1.12 | 0.62 | 2.03 | 0.71 | 1.30 | 0.53 | 3.19 | 0.57 | 1.02 | 0.53 | 1.93 | 0.96 | 1.08 | 0.55 | 2.13 | 0.82 | 0.85 | 0.24 | 2.98 | 0.80 |
| Hardship financing vs none | 1.19 | 0.70 | 2.04 | 0.52 | 1.04 | 0.50 | 2.17 | 0.91 | 1.27 | 0.71 | 2.28 | 0.42 | 1.42 | 0.78 | 2.57 | 0.25 | 1.56 | 0.56 | 4.35 | 0.40 |
| Travel time ≥ 1 hr vs < 1hr | 1.02 | 0.59 | 1.76 | 0.94 | 1.90 | 0.91 | 3.96 | 0.09 | 0.43 | 0.24 | 0.76 | 0.003* | 0.52 | 0.29 | 0.94 | 0.03* | 0.45 | 0.13 | 1.61 | 0.22 |
| 2013 | 1.00 |  |  |  | 1.00 |  |  |  | 1.00 |  |  |  | 1.00 |  |  |  | 1.00 |  |  |  |
| 2014 | 0.78 | 0.46 | 1.32 | 0.36 | 6.42 | 3.08 | 13.37 | <0.001* | 0.35 | 0.20 | 0.62 | <0.001* | 0.42 | 0.23 | 0.74 | 0.003* | 2.49 | 0.88 | 6.99 | 0.08 |
| 2015 | 4.63 | 1.03 | 20.87 | 0.05* | 1.88 | 0.29 | 12.40 | 0.51 | 1.68 | 0.44 | 6.45 | 0.45 | 4.65 | 0.57 | 37.77 | 0.15 | 2.18 | 0.17 | 28.04 | 0.55 |

Legend: statistically significant p values <0.05 are marked with an asterisk *

# Supplemental Table 6: Multivariable logistic regression on poverty and distance factors, with only MPI poverty, associated with visit adherence measures.

|  | Visit Constancy | | | | No Gaps in Care | | | | Visit in Last Quarter | | | | At least 6 visits per year | | | | Yes on all metrics | | | |
| --- | --- | --- | --- | --- | --- | --- | --- | --- | --- | --- | --- | --- | --- | --- | --- | --- | --- | --- | --- | --- |
| Characteristic | OR | 95% CI | | p value | OR | 95% CI | | p value | OR | 95% CI | | p value | OR | 95% CI | | p value | OR | 95% CI | | p value |
| Female vs male | 0.78 | 0.47 | 1.28 | 0.32 | 0.79 | 0.44 | 1.44 | 0.45 | 1.7 | 1.03 | 2.82 | 0.04* | 1.69 | 1.01 | 2.83 | 0.05* | 0.66 | 0.33 | 1.32 | 0.24 |
| Age (10 year increments) | 1.14 | 0.97 | 1.33 | 0.1 | 1.27 | 1.04 | 1.54 | 0.02* | 0.78 | 0.67 | 0.92 | <0.001* | 0.84 | 0.7 | 1.01 | 0.06 | 1.1 | 0.88 | 1.37 | 0.42 |
| Poor | 1.26 | 0.79 | 2.01 | 0.34 | 1.05 | 0.58 | 1.89 | 0.88 | 0.85 | 0.52 | 1.39 | 0.52 | 1.13 | 0.67 | 1.91 | 0.64 | 0.82 | 0.4 | 1.72 | 0.61 |
| Euclidean distance (10 km) | 1.03 | 0.93 | 1.14 | 0.56 | 1.05 | 0.95 | 1.17 | 0.32 | 0.90 | 0.81 | 0.99 | 0.04* | 0.95 | 0.86 | 1.05 | 0.33 | 0.85 | 0.71 | 1.03 | 0.10 |
| 2013 | 1 |  |  |  | 1 |  |  |  | 1 |  |  |  | 1 |  |  |  | 1 |  |  |  |
| 2014 | 0.95 | 0.6 | 1.51 | 0.83 | 8.04 | 4.07 | 15.92 | <0.001* | 0.4 | 0.25 | 0.66 | <0.001* | 0.53 | 0.32 | 0.88 | 0.01* | 3.96 | 1.67 | 9.38 | <0.001* |
| 2015 | 5.94 | 2.04 | 17.31 | <0.001* | 8.14 | 3.02 | 21.95 | <0.001* | 1.42 | 0.52 | 3.86 | 0.49 | 2.93 | 0.83 | 10.4 | 0.1 | 11.77 | 3.79 | 36.53 | <0.001* |

Legend: statistically significant p values <0.05 are marked with an asterisk *

# Supplemental Table 7: Multivariable logistic regression on poverty and distance factors, with only hardship financing, associated with visit adherence measures.

|  | Visit Constancy | | | | No Gaps in Care | | | | Visit in Last Quarter | | | | At least 6 visits per year | | | | Yes on all metrics | | | |
| --- | --- | --- | --- | --- | --- | --- | --- | --- | --- | --- | --- | --- | --- | --- | --- | --- | --- | --- | --- | --- |
| Characteristic | OR | 95% CI | | p value | OR | 95% CI | | p value | OR | 95% CI | | p value | OR | 95% CI | | p value | OR | 95% CI | | p value |
| Female vs male | 0.87 | 0.51 | 1.51 | 0.62 | 0.81 | 0.4 | 1.66 | 0.57 | 2.30 | 1.30 | 4.07 | <0.001* | 2.04 | 1.15 | 3.63 | 0.02* | 0.91 | 0.35 | 2.35 | 0.85 |
| Age (10 year increments) | 1.11 | 0.94 | 1.32 | 0.22 | 1.34 | 1.08 | 1.67 | 0.01* | 0.71 | 0.59 | 0.85 | <0.001* | 0.81 | 0.66 | 0.99 | 0.04* | 1.15 | 0.9 | 1.47 | 0.26 |
| Hardship financing vs none | 1.13 | 0.67 | 1.89 | 0.65 | 1.08 | 0.53 | 2.17 | 0.84 | 1.29 | 0.74 | 2.25 | 0.37 | 1.35 | 0.77 | 2.39 | 0.3 | 1.41 | 0.53 | 3.74 | 0.49 |
| Euclidean distance (10 km) | 1.02 | 0.92 | 1.12 | 0.75 | 1.08 | 0.96 | 1.21 | 0.19 | 0.87 | 0.78 | 0.98 | 0.03* | 0.93 | 0.84 | 1.03 | 0.16 | 0.80 | 0.59 | 1.09 | 0.16 |
| 2013 | 1 |  |  |  | 1 |  |  |  | 1 |  |  |  | 1 |  |  |  | 1 |  |  |  |
| 2014 | 0.77 | 0.46 | 1.29 | 0.32 | 7.18 | 3.46 | 14.87 | <0.001* | 0.34 | 0.19 | 0.59 | <0.001* | 0.39 | 0.22 | 0.69 | <0.001* | 3.05 | 1.15 | 8.11 | 0.03* |
| 2015 | 4.81 | 1.06 | 21.79 | 0.04* | 3.03 | 0.71 | 12.92 | 0.13 | 1.06 | 0.31 | 3.65 | 0.93 | 3.91 | 0.49 | 30.85 | 0.2 | 4.83 | 0.94 | 24.8 | 0.06 |

Legend: statistically significant p values <0.05 are marked with an asterisk *

# Supplemental Table 8: Multivariable logistic regression on disease severity, associated with visit adherence measures.

|  | Visit Constancy | | | | No Gaps in Care | | | | Visit in Last Quarter | | | | At least 6 visits per year | | | | Yes on all metrics | | | | |
| --- | --- | --- | --- | --- | --- | --- | --- | --- | --- | --- | --- | --- | --- | --- | --- | --- | --- | --- | --- | --- | --- |
| Characteristic | OR | 95% CI | | p value | OR | 95% CI | | p value | OR | 95% CI | | p value | OR | 95% CI | | p value | OR | 95% CI | | p value |  |
| Female vs male | 0.91 | 0.59 | 1.40 | 0.66 | 1.05 | 0.60 | 1.83 | 0.86 | 1.45 | 0.91 | 2.30 | 0.12 | 1.84 | 1.16 | 2.95 | 0.01 | 0.87 | 0.43 | 1.73 | 0.68 |  |
| Age (10 year increments) | 1.13 | 0.98 | 1.30 | 0.09 | 1.25 | 1.05 | 1.48 | 0.01 | 0.80 | 0.69 | 0.93 | 0.00 | 0.92 | 0.78 | 1.08 | 0.30 | 1.06 | 0.86 | 1.29 | 0.60 |  |
| Other diagnoses | 1.00 |  |  |  | 1.00 |  |  |  | 1.00 |  |  |  | 1.00 |  |  |  | 1.00 |  |  |  |  |
| Diabetes Type I | NA |  |  |  | 8.81 | 1.06 | 73.13 | 0.04 | 0.31 | 0.02 | 4.27 | 0.38 | 0.34 | 0.02 | 4.73 | 0.42 | NA |  |  |  |  |
| Type II on insulin | 1.46 | 0.75 | 2.84 | 0.27 | 2.58 | 1.20 | 5.56 | 0.02 | 0.81 | 0.41 | 1.59 | 0.53 | 1.68 | 0.77 | 3.67 | 0.19 | 2.37 | 0.97 | 5.79 | 0.06 |  |
| Congestive Heart Failure | 1.00 |  |  |  | 3.07 | 1.22 | 7.71 | 0.02 | 0.52 | 0.22 | 1.22 | 0.13 | 1.75 | 0.68 | 4.51 | 0.24 | 1.47 | 0.41 | 5.18 | 0.55 |  |
| 2013 | 1.00 |  |  |  | 1.00 |  |  |  | 1.00 |  |  |  | 1.00 |  |  |  | 1.00 |  |  |  |  |
| 2014 | 1.25 | 0.84 | 1.88 | 0.27 | 7.97 | 4.14 | 15.37 | 0.00 | 0.40 | 0.26 | 0.62 | 0.00 | 0.60 | 0.39 | 0.94 | 0.03 | 3.35 | 1.49 | 7.54 | 0.00 |  |
| 2015 | 4.60 | 2.22 | 9.52 | 0.00 | 7.67 | 3.44 | 17.08 | 0.00 | 1.09 | 0.53 | 2.24 | 0.80 | 3.53 | 1.35 | 9.24 | 0.01 | 6.38 | 2.60 | 15.61 | 0.00 |  |

# Supplemental Table 9: Multivariable logistic regression on poverty and distance factors, adjusting for disease severity, associated with visit adherence measures.

|  | Visit Constancy | | | | No Gaps in Care | | | | Visit in Last Quarter | | | | At least 6 visits per year | | | | Yes on all metrics | | | |
| --- | --- | --- | --- | --- | --- | --- | --- | --- | --- | --- | --- | --- | --- | --- | --- | --- | --- | --- | --- | --- |
| Characteristic | OR | 95% CI | | p value | OR | 95% CI | | p value | OR | 95% CI | | p value | OR | 95% CI | | p value | OR | 95% CI | | p value |
| Female vs male | 0.92 | 0.52 | 1.63 | 0.78 | 0.93 | 0.45 | 1.93 | 0.85 | 2.31 | 1.27 | 4.22 | 0.01 | 2.36 | 1.28 | 4.37 | 0.01 | 1.18 | 0.44 | 3.19 | 0.75 |
| Age (10 year increments) | 1.14 | 0.95 | 1.37 | 0.15 | 1.50 | 1.18 | 1.91 | 0.00 | 0.71 | 0.58 | 0.87 | 0.00 | 0.84 | 0.67 | 1.06 | 0.15 | 1.33 | 1.03 | 1.71 | 0.03 |
| Poor | 1.12 | 0.61 | 2.05 | 0.71 | 1.14 | 0.45 | 2.85 | 0.78 | 1.03 | 0.55 | 1.94 | 0.92 | 1.03 | 0.52 | 2.05 | 0.92 | 0.89 | 0.25 | 3.12 | 0.86 |
| Hardship financing vs none | 1.11 | 0.65 | 1.89 | 0.71 | 1.10 | 0.53 | 2.25 | 0.80 | 1.28 | 0.73 | 2.26 | 0.39 | 1.31 | 0.73 | 2.36 | 0.36 | 1.50 | 0.57 | 3.97 | 0.41 |
| Euclidean distance (10 km) | 1.09 | 0.96 | 1.23 | 0.17 | 1.05 | 0.92 | 1.20 | 0.49 | 0.87 | 0.76 | 0.98 | 0.03 | 0.91 | 0.80 | 1.03 | 0.14 | 0.79 | 0.58 | 1.07 | 0.13 |
| Other diagnoses | 1.00 |  |  |  | 1.00 |  |  |  | 1.00 |  |  |  | 1.00 |  |  |  | 1.00 |  |  |  |
| Diabetes Type I | NA |  |  |  | 5.72 | 0.43 | 76.57 | 0.19 | 1.68 | 0.17 | 16.36 | 0.65 | 0.92 | 0.09 | 9.52 | 0.95 | NA |  |  |  |
| Type II on insulin | 1.91 | 0.78 | 4.70 | 0.16 | 4.26 | 1.65 | 11.02 | 0.00 | 1.03 | 0.42 | 2.55 | 0.95 | 2.44 | 0.83 | 7.20 | 0.10 | 5.04 | 1.61 | 15.75 | 0.01 |
| Congestive Heart Failure | 0.66 | 0.22 | 1.98 | 0.46 | 1.63 | 0.35 | 7.58 | 0.54 | 1.16 | 0.34 | 3.94 | 0.81 | 2.87 | 0.64 | 12.90 | 0.17 | 1.48 | 0.14 | 15.45 | 0.74 |
| 2013 | 1.00 |  |  |  | 1.00 |  |  |  | 1.00 |  |  |  | 1.00 |  |  |  | 1.00 |  |  |  |
| 2014 | 0.73 | 0.43 | 1.23 | 0.24 | 8.30 | 3.77 | 18.27 | 0.00 | 0.34 | 0.19 | 0.59 | 0.00 | 0.39 | 0.22 | 0.70 | 0.00 | 3.45 | 1.24 | 9.58 | 0.02 |
| 2015 | 4.65 | 1.06 | 20.51 | 0.04 | 3.26 | 0.72 | 14.86 | 0.13 | 1.07 | 0.30 | 3.81 | 0.91 | 3.57 | 0.47 | 26.87 | 0.22 | 4.94 | 0.95 | 25.77 | 0.06 |

Legend: statistically significant p values <0.05 are marked with an asterisk *

# Supplemental Figure 1: Flow diagram of database linkage, and patient selection


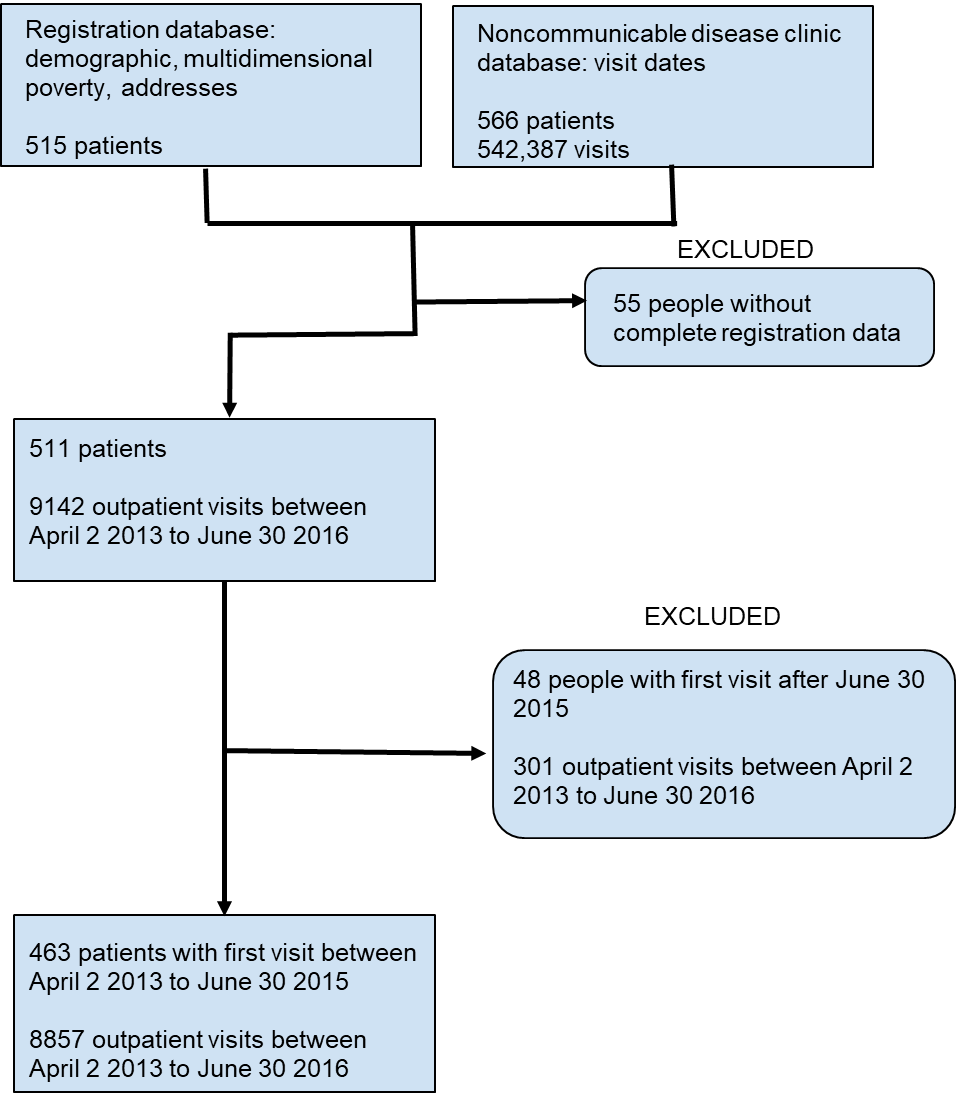


# Supplemental Figure 2: Density map of visit adherence measures.


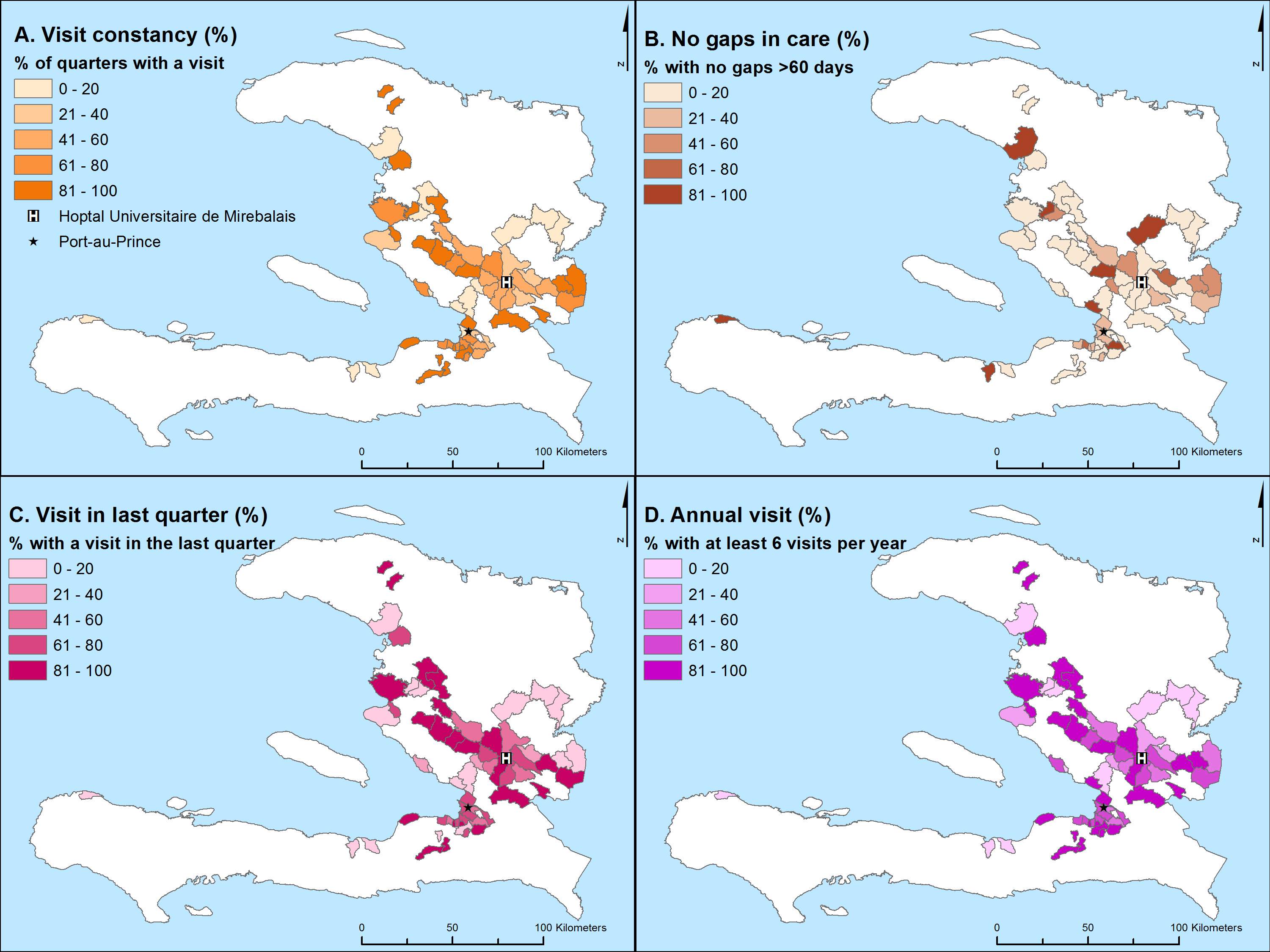


Legend: Darker colors indicate higher density of either patient residence (absolute numbers) or good visit adherence on all four metrics (percentage). The location of the capital Port-au-Prince, and the Hopital Universitaire de Mirebalais are labeled.

# Supplemental Figure 3: Odds ratios for factors related to visit adherence measure outcomes.
